# State-of-the-Art Review of Pregnancy-Related Psoriasis

**DOI:** 10.3390/medicina57080804

**Published:** 2021-08-05

**Authors:** Anca Angela Simionescu, Bianca Mihaela Danciu, Ana Maria Alexandra Stanescu

**Affiliations:** 1 Department of Obstetrics and Gynecology, Filantropia Clinical Hospital, Carol Davila University of Medicine and Pharmacy, 050474 Bucharest, Romania; 2 Department of Obstetrics, Gynecology and Neonatology, “Dr. Alfred Rusescu” National Institute for Maternal and Child Health, 127715 Bucharest, Romania; biamidan@yahoo.com; 3 Department of Family Medicine, Carol Davila University of Medicine and Pharmacy, 050474 Bucharest, Romania

**Keywords:** psoriasis, pregnancy, high-risk pregnancy, hypertensive disorders, gestational diabetes, low birth weight

## Abstract

Psoriasis is a chronic immunologic disease involving inflammation that can target internal organs, the skin, and joints. The peak incidence occurs between the age of 30 and 40 years, which overlaps with the typical reproductive period of women. Because of comorbidities that can accompany psoriasis, including metabolic syndrome, cardiovascular involvement, and major depressive disorders, the condition is a complex one. The role of hormones during pregnancy in the lesion dynamics of psoriasis is unclear, and it is important to resolve the implications of this pathology during pregnancy are. Furthermore, treating pregnant women who have psoriasis represents a challenge as most drugs generally prescribed for this pathology are contraindicated in pregnancy because of teratogenic effects. This review covers the state of the art in psoriasis associated with pregnancy. Careful pregnancy monitoring in moderate-to-severe psoriasis vulgaris is required given the high risk of related complications in pregnancy, including pregnancy-induced hypertensive disorders, low birth weight for gestational age, and gestational diabetes. Topical corticosteroids are safe during pregnancy but effective only for localised forms of psoriasis. Monoclonal antibodies targeting cytokines specifically upregulated in psoriasis, such as ustekinumab (IL-12/23 inhibitor), secukinumab (IL-17 inhibitor) can be effective for the severe form of psoriasis during pregnancy. A multidisciplinary team must choose optimal treatment, taking into account fetal and maternal risks and benefits.

## 1. Introduction

Psoriasis is a chronic inflammatory disease, a T cell–mediated disorder secondary to inflammation and keratinocyte hyperproliferation that affects 1–3% of the population [1]. Its course is unpredictable and capricious but usually is associated with chronic immune-mediated findings and generalised inflammatory disease. Most forms begin before the age of 40, which corresponds with the reproductive period for most women [2,3]. Psoriasis is associated with comorbidities such as metabolic syndrome (diabetes mellitus, arterial hypertension), cardiovascular diseases, other autoimmune disorders, depressive disorders, and cancer [1,4,5,6,7]. The prevalence of psoriasis in women of reproductive age is approximately 1% [8]. Genetic studies have reported pregnancy as predisposition to severe generalised pustular psoriasis for persons carrying the mutation of the interleukin 36 receptor antagonist gene (IL36RN genes) [9]. Also HLA-C alleles [10,11] and SNPs/mutations in genes play an important role in inflammatory processes mechanisms that influence the immune response or the differentiation of keratinocytes and immune cells (e.g., CARD14, IL23R, IL12B) [12,13]. Individuals carrying HLA-Cw* 0602 have about a 10-fold increased relative risk, and those who are homozygous for this allele have a more than a 20-fold increased risk of developing psoriasis. HLA-Cw* 0602 positive patients are predisposed to an earlier age onset on lesions than those who are negative [11]. The genetic predisposition may play a role in antigen-specific activation and differentiation of T-cell mediated autoimmune disease [14].

Given that the evolution and severity of psoriasis during pregnancy cannot be predicted, optimal treatment choices need to be individualised. Pregnant women can have improved [8,15] or worsened outcomes during pregnancy, mainly because of specific immunologic adaptations during pregnancy and the lack of medication changes. Most women with more than one pregnancy who experienced improvement during earlier pregnancies report a similar response during subsequent gestations [16]. However, there are cases of a different clinical disease evolution across pregnancies and postpartum periods in the same individual [17]. Improvement during pregnancy has been reported for women who carry HLA-Cw* 0602, but women with psoriasis who do not carry this allele tend to experience unchanged or worsening disease [11]. During the postpartum period, it is common for psoriasis to worsen in the form of new skin lesions or an extension of existing lesions [18,19].

The risks of an infant developing psoriasis are 50% if both parents have psoriasis, 16% if one parent has psoriasis, and 8% if neither parent but one sibling has psoriasis [20].

The implications of immune tolerance in psoriasis-related to maternal-fetal immune tolerance during pregnancy, and how pregnancy-specific hormones influence the T cell cytokines responses involved in the evolution of the disease are intensely debated topics of multidisciplinary interest. Contrary to pregnancy-associated breast cancer (P.A.B.C.) when P.A.B.C. is associated with prognosis factors similar to those reported among breast cancer (B.C.) patients over maternal age [21,22], pregnancy may affect the expression of autoimmune disease and autoimmune disease and cytokines imbalance may interfere with trophoblast and placental development [8,23]. Also, dendritic and T cells” immune response to fetal semi-allograft are much more complicated than their response after the transplanted allograft [24].

The pathogenesis of psoriasis lesions is attributed to the dysfunction of T-cell subsets including T-helper (Th) 1 cells, Th2, Th17, Th22 and regulatory T cells (Tregs) and the resulting aberrant release of the corresponding cytokines including IFN-γ, tumour necrosis factor (TNF)-α, IL-23 and IL-17 family members [25,26]. The immunology of pregnancy is related to CD4+ T cell cytokines and T cell responses in autoimmune disease are influenced by pregnancy. There are immune shifts in pregnancy to facilitate maternal-fetal tolerance, the maternal immune response changes from the inflammatory Th1 cytokine pattern to the Th2 pattern [27]. Regulatory T cells (Tregs) play an essential role in immune homeostasis by suppressing immune responses. Tregs are impaired in their suppressive function in psoriasis, leading to an altered T-helper 17/Treg balance [25]. In pregnancy, the maintenance of pregnancy is related to Th2 and Th17/Th2 cells and Treg cells. Th-1-type and Th17-type cytokines that promote fetal semi-allograft rejection may compromise pregnancy, whereas Th2-type cytokines may improve pregnancy outcomes [28,29,30].

Moreover, treating pregnant women with psoriasis represents a challenge as most generally prescribed drugs are contraindicated in pregnancy because of their teratogenic effects.

Here, we review the current state of the art to construct a comprehensive but straightforward assessment of psoriasis during pregnancy. We emphasise both the difficult treatment decisions during the pregnancy and postpartum periods and the high-risk pregnancy complications arising from psoriasis-associated pathologies. With this approach, we hope to raise awareness for obstetricians, general practitioners, dermatologists, and others who may be involved in monitoring these cases.

## 2. Clinical Phenotypes of Psoriasis in Pregnancy

Psoriasis has the following clinical presentations that have been circumscribed as specific phenotypes [1].

**Figure 1:**
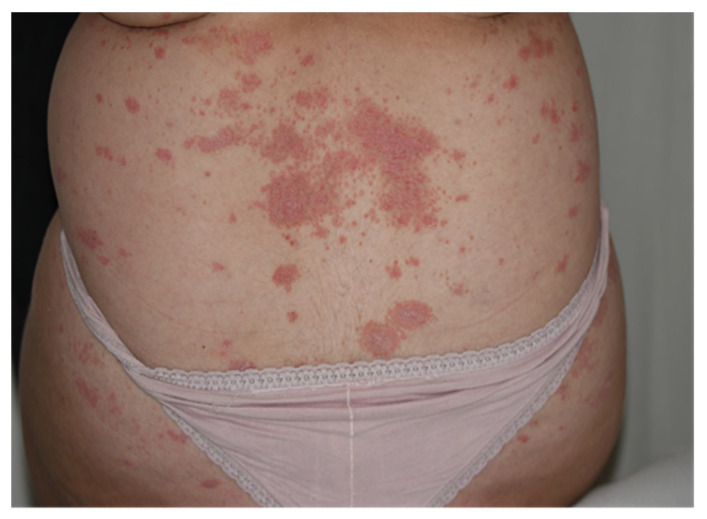
Psoriasis Vulgaris after use of keratolytic agents: skin plaques located on the lower back.

Plaque psoriasis (Psoriasis Vulgaris), the most common form, is characterised by red or salmon pink papulosquamous skin plaques covered by white or silvery scales and well-delineated from surrounding normal skin. The plaques are distributed symmetrically and arise most commonly on the extensor aspects of elbows and knees (Figure 1).

Guttate psoriasis—some adolescents who become pregnant can develop an acute, self-limiting form of psoriasis known as guttate psoriasis (from the Latin *gutta*, meaning ‘droplet’). In this form of psoriasis, papules less than 1 cm in diameter erupt on the trunk about 2 weeks after a β-hemolytic streptococcal infection such as tonsillitis or pharyngitis, or a viral infection.

**Figure 2:**
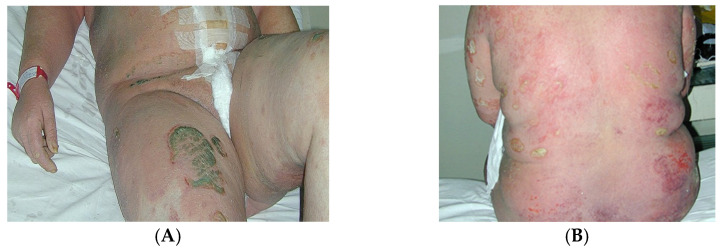
(**A**) Patient 24 h postpartum with generalised pustular psoriasis (von Zumbusch psoriasis). (**B**) Detail—the pustules are sterile and amicrobial, formed through an immune mechanism.

Von Zumbusch psoriasis (generalised pustular psoriasis) is an acute and painful inflammatory form of the disease characterised by small, monomorphic sterile pustules (Figure 2A,B) associated with systemic symptoms.

Erythrodermic psoriasis is characterised by papulosquamous skin plaques on the whole body surface. This type of psoriasis can be a life-threatening condition that leads to hypothermia, hypoalbuminemia, and high output cardiac failure.

Psoriatic arthritis is a seronegative inflammatory arthritis that occurs in the presence of cutaneous lesions. The timing of the association between articular and skin lesions is variable, and skin involvement usually manifests first.

**Figure 3:**
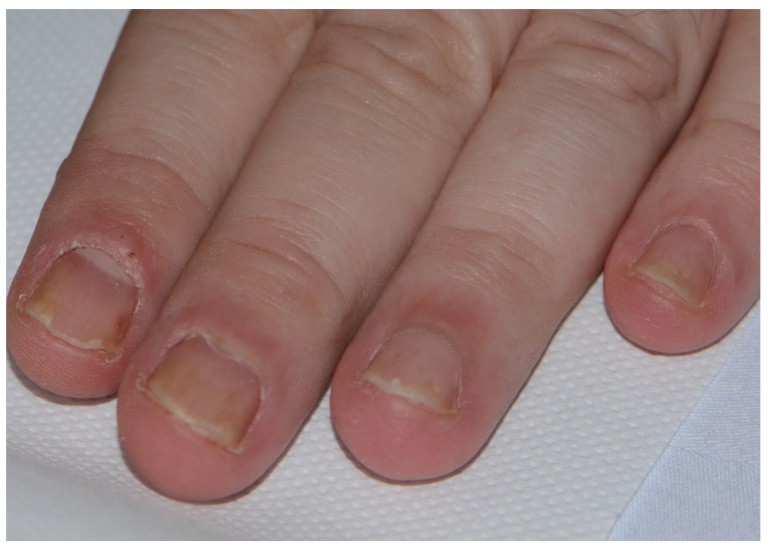
Psoriatic nail dystrophy.

Specific nail changes related to psoriasis nail dystrophy include the following: the presence of pitting, which is best seen under oblique lighting conditions; onycholysis (nail plate separation); oil spots (orange-yellow subungual discolouration); and dystrophic nails, similar to that observed in onychomycosis (Figure 3). Psoriatic nail disease occurs most commonly in patients with psoriatic arthritis.

Specific nail changes related to psoriasis can be described as-nail matrix involvement which is reflected in: involvement of the proximal matrix produces pitting, Beau’s lines, nail onychomadesis leading to nail loss and trachionichia, while the involvement of the intermediate matrix is responsible for leukonychia. Involvement of the subungual tissues distal to the lunula results in subungual hyperkeratosis, onycholysis, splinter haemorrhages or oily spots [31]. The whole nail unit may be affected by psoriasis. Nail changes are similar to that observed in onychomycosis (Figure 3). Psoriatic nail disease occurs most commonly in patients with psoriatic arthritis.

Pustular psoriasis of pregnancy (P.P.P., impetigo herpetiformis) is considered a variant of pustular psoriasis, a severe generalised pustular psoriasis occurring in pregnancy regardless of trimester. However, it is a rare dermatosis occurring during the third trimester of pregnancy and followed by a resolution in the postpartum period. P.P.P. is characterised by generalised pruritic rash, sterile pustules scattered on erythematous patches within intertriginous areas (under the breast, axillae skin fold) centrifugally spread to the extremities. Because it is considered a high-risk dermatosis with maternal and fetal consequences, it is important to be early recognised and treated [32,33,34].

The Psoriasis Area and Severity Index (P.A.S.I.) score is the best-validated score used to evaluate the clinical severity of skin psoriasis [35].

## 3. Evolution of Psoriasis Activity during Pregnancy

Psoriasis is one of the most common chronic immune-mediated pathologies. Tumour necrosis factor (TNF)-alpha, dendritic cells, and T cells contribute substantially to the immune pathogenesis of this disease [1]. Immunologic requirements for maternal tolerance toward the fetus and the immunologic reset to a non-pregnant state influence disease evolution during pregnancy, including maternal immune and autoimmune diseases. Many reviews have shown that the evolution of psoriasis is unpredictable during pregnancy [36]. Also, major susceptibility gene for psoriasis on chromosome 6 may be related to the clinical phenotype, the age for the onset, and evolution [11].

Table 1 and Table 2 present the clinical evolution of psoriasis activity in pregnant women, as described in the medical literature, independent of treatment received or severity of psoriasis. Most cases were evaluated based on self-evaluation and questionnaires. We note clinical evaluations reported during the second and third trimesters of pregnancy and up to one year postpartum. Skin improvement was considered when authors reported a reduction in P.A.S.I. score or when patients’ assessments were “improved”. Joint activity improvement was considered when authors reported improvement based on a scale evaluation (e.g., the Bath Ankylosing Spondylitis Disease Activity Index (B.A.S.D.A.I.) or based on patients’ auto-evaluation [37].

Other clinical domains included in composite measures in PsA and clinical assessment of psoriasis outcome measures are shown in Table 3.

Despite the small number of patients, in more than 40% of cases, psoriasis improved during pregnancy, compared with almost 20% showing a worse evolution and 21% to 56% remaining stable. More than half of cases worsened in the postpartum period. In patients who were Cw* 0602 carriers, an inverse relationship was observed between the age of onset and disease severity [11]. In patients who were not Cw* 0602 carriers, age of onset was positively associated with disease severity score.

This clinical evolution could be explained by changes resulting from the interaction between hormonal fluctuations during pregnancy and the immune system. T cell responses in autoimmune disorders are influenced by pregnancy, and hormonal changes during pregnancy could play a critical role in determining the effector CD4+ T cell cytokine profile at the fetomaternal interface. T helper (Th) cells are classified as Th1, Th2, and Th17 based on cytokines they produce.

CD4+ Th1 cells produce interleukin (IL)-2, TNF-β, and interferon (I.F.N.)-γ and are the main effectors of phagocyte-mediated host defence. CD4 + Th1 cells are highly protective against intracellular pathogen infections [19]. CD4+ Th2 cells are mainly responsible for phagocyte-independent host defense against extracellular pathogens. CD4+ Th cells, the Th17 cells, produce IL-17A, IL-17F, IL-21, IL-26, and IL-22. CD4+ Th17 is protective against extracellular bacteria and may also play a role in inflammation [51]. Hormone and Th cell changes also could explain why, in some conditions, pregnant women are more prone to developing Th2-type autoimmune diseases, whereas the Th1- and Th17-type autoimmune and inflammatory diseases are improved [30].

Psoriasis pathogenesis is induced by melanocyte presentation of autoantigens to autoreactive epidermal CD8+ T cells, which determine increased levels of TNF-α, IL-17, and Il-17/IL-23. These cytokines stimulate keratinocyte proliferation, resulting in acanthosis and para- and hyperkeratosis, which in turn leads to the formation of microabscesses and plaques within the stratum corneum [52].

The current theory of psoriasis is that the dendritic cell–Th17/IL23 axis is the main axis of the disease. In psoriasis, IL-23 is overproduced by dendritic cells and keratinocytes, and this stimulates Th17 cells within the dermis to make IL-17A and IL-22. IL-22, in particular, is responsible for keratinocyte hyperproliferation [53]. Also, epidermal keratinocytes express high amounts of antimicrobial peptides (A.M.P.s), in particular, the cathelicidin LL-37 with immunomodulatory activity, its aberrant expression can lead to uncontrolled inflammation in autoimmune diseases, including psoriasis. In the development of psoriasis, keratinocytes overproduce several innate immunity mediators, including IL-1 cytokines, chemokines, and A.M.P.s, particularly LL-37, HBD-2, and psoriasin [54]. In pregnancy, the exact role of cathelicidin is unknown but was related to modulating inflammatory processes [55].

During pregnancy, women experience increased levels of glucocorticoid concentrations to support this immunosuppressive status. Compared to pre-pregnancy, these levels approximately double during the third trimester and at 2 and 3 months postpartum [56,57]. Human placental lactogen, prolactin, and human chorionic gonadotropin also have high values during pregnancy and contribute to immunosuppressive status [58]. During pregnancy, the cellular immune response shows a decrease in the number of cells positive for CD3, CD4, CD8, and CD20 and an increase in cells positive for CD4, CD16, and CD20 in the first month in postpartum and CD3 and CD56 at 4 months postpartum [59].

High estrogen levels cause this switch between the two types of immunity (Th1 and Th2) during pregnancy. To support this claim, even non-pregnant patients with psoriasis who are taking high-dose estrogen oral contraceptives have experienced an improvement in the course of their disease. Conversely, patients who are postpartum or in menopause experience an unfavorable evolution of psoriatic disease. Estrogens have been shown to have a role in autoantibody production, and progesterone is actively involved in the regulation of the immune response [60].

The only form of psoriasis that shows unfavourable evolution in most cases during pregnancy, including fetal and maternal morbidity and mortality, is P.P.P. (impetigo herpetiformis) [61]. Impetigo herpetiformis tends to remain active until birth; after birth under treatment the lesions tend to disappear, very rarely it is present in the postpartum period. A particular problem is represented by systemic complications that tend to occur such as an increase in leukocytes and neutrophils, fever, tetany, hypoproteinemia, decrease in serum calcium levels, and in severe cases erosion of mucous membranes over the tongue, oral cavity, and esophagus [62]. A case was presented that did not respond to treatment that required termination of pregnancy at the gestational age of 20 weeks. The patient fully recovered three months after the termination of pregnancy [63].

## 4. Specific Complications of Pregnancy-Associated Psoriasis

Pregnancy in women with psoriasis represents a high-risk situation because of the limited therapeutic options and multiple comorbidities [5,64]. Moderate-to-severe psoriasis is associated during pregnancy with pregnancy-induced hypertensive disorders, low birth weight (L.B.W.) for gestational age, preterm birth (PTB), depressive disorders, and gestational diabetes [5] and even spontaneous abortion [4,24,65]. In these cases, psoriasis-specific pro-inflammatory cytokines such as IL-1, IL-6, and TNF-alpha occur at much higher levels than usual in maternal serum or umbilical cord blood [66,67,68]. In L.B.W. and PTB, levels of pro-inflammatory cytokines are increased in maternal serum and umbilical cord blood. These are the same pro-inflammatory cytokines that underlie the immune mechanisms of psoriasis, including TNF-alpha, IL-6, and C-reactive protein [68,69]. These excess cytokines cause a rise in placental vasculopathy, ultimately explaining L.B.W. [68,69].

Associated pathologies such as anxiety and depressive disorders, metabolic syndrome (diabetes, obesity, hypertension), cardiovascular disease, vitamin D deficiency, inflammatory bowel disease, and psoriatic or inflammatory arthritis are also more commonly reported for severe forms of psoriasis [1,4,5,6,7]. Maternal alcohol and tobacco consumption were reported frequently associated with the severity of psoriasis [70].

In a recent systematic review on 4756 pregnancy outcomes, Bobotsis et al. found an inconsistent relationship between psoriasis and spontaneous abortion, L.B.W., macrosomia, and prematurity [71].

Because pregnant women cannot receive systemic treatment and treatment is discontinued during pregnancy, worsening and aggravation of psoriasis both occur, especially postpartum [72]. There is a statistically significant aggravation of lesions during the postpartum period [4,37,73].

In a case-control study on pregnancy outcomes in 35 women with moderate-to-severe psoriasis in Israel, Cohen-Barak et al. found a higher proportion of spontaneous abortion. Of the 68 deliveries, the authors identified a significant proportion of spontaneous abortion and premature birth. Birth weight >4000 g was reported in 13.4% of cases, and birth weight less <2500 g was reported in 7.4%, all probably at term [4]. Harder et al. reported no increased number of cases with diabetes or hypertension in a Danish population [74].

In a study of 162 pregnancies associated with psoriasis, Lima et al. reported a 1.89-fold increased odds of poor outcome (95% CI 1.06–3.39), but no statistically significant risk for L.B.W., PBW, or preeclampsia [24].

P.P.P. is considered a high-risk dermatosis of pregnancy for both mother and fetus. A typical maternal eruption is associated with systemic symptoms of fever and malaise and may lead to the loss of fluids and electrolytes disorders and maternal sepsis. Therefore, skin biopsy and skin cultures should be performed. Due to the high risk of fetal complications, patients with P.P.P. must be carefully monitored to decide optimal delivery time [33,34,75].

## 5. Treatment of Psoriasis during Pregnancy

The classical treatment regimens for psoriasis include topical and systemic therapies and, recently, human monoclonal antibodies for moderate to severe forms. These treatments include antagonists of IL-17 or IL-23, such as secukinumab or ustekinumab [20].

Preconception counselling and timing of conception for when psoriasis is controlled or in remission are important issues for a multidisciplinary team to address. Many authors agree that controlling disease activity prior to conception may lead to less psoriasis activity during pregnancy [17,20].

Although pregnant women cannot be included in clinical trials, information regarding psoriasis therapy, mainly during the first week of pregnancy, is available from participants who were unaware of their pregnancy and responded to questionnaires [36,76,77].

Topical treatment is the first line in treating psoriasis during pregnancy when respecting the optimal doses [36,78]. In general, drugs with minimal systemic absorption are chosen to minimise possible teratogenic effects. The most indicated are emollient creams, moisturizers, tacrolimus, dermatocorticosteroids, and tazarotene.

Tacrolimus crosses into the fetal circulation and maternal breast milk and has been found in maternal blood and venous umbilical cord. It is associated with L.B.W., prematurity, developmental thymus dysfunction, potential alterations in fetal brain development, transient neonatal hyperkalemia, and renal dysfunction [79,80,81,82,83]. A recent report of 15 infants breastfed while their mothers were taking systemic tacrolimus did not find high concentrations in infants or any effect on the infants [80].

Topical corticosteroids are safe during pregnancy but effective only for localised forms of psoriasis [84]. High doses are associated with L.B.W. [84]. Topical corticosteroid use in the postpartum period also has been implicated in the formation of striae on the breasts during breastfeeding [85] and should be avoided in this region. 

In 2658 patients from a case-control study, mild to moderate topical corticosteroids <300 g were safe during pregnancy. Moreover, this study included pregnant women with autoimmune diseases in whom the underlying disease caused placental insufficiency and other obstetric complications. For 757 pregnant women, corticosteroid administration was in the first trimester. When potent or very potent topical corticosteroids are needed, the administered doses should be kept to a minimum [79,84].

Although the topical retinoid tazarotene reaches a relatively low maternal concentration and no maternal and fetal complications were associated with it in case of accidental exposure, the U.S. Food and Drug Administration (F.D.A.) lists this drug as a category C [86].

As a second line, UVB phototherapy may be safely used by itself or in combination with other topical treatments [77,78]. For generalised psoriasis, UVB represents the best option during pregnancy [20]. This therapy is considered safe as long as the cumulative dose is not high. When the cumulative dose exceeds the threshold, a decline in folic acid levels has been reported [87,88,89]. Thus, the recommendation is to supplement with folic acid at 4–5 mg/day during UVB therapy to avoid neural tube malformations. Patients may report worsening melasma with UVB therapy [78].

Psoralen plus UVA (P.U.V.A. therapy) during pregnancy is not associated with a significantly increased incidence of fetal malformations [77,78,90] but is linked to an increased rate of L.B.W. Because psoralen triggers photosensitivity, its use is not recommended during pregnancy. Most patients stop the treatment early in pregnancy. Severe disease with no available treatment or a non-optimal therapeutic response may negatively affect both the mother and fetus [2,91].

Third-line systemic therapies are indicated for severe psoriasis after the information is provided to patients about side effects for the mother and fetus. The F.D.A. classifies these drugs as class C. Systemic therapies used during pregnancy are cyclosporin, systemic corticosteroids, and TNF-alpha inhibitors.

Cyclosporine A crosses the fetoplacental barrier and may be correlated with prematurity and L.B.W. [92]. This drug passes into breast milk at high concentrations, and there is a risk of induced neonatal immunosuppression [93,94]. Cyclosporine can also be tried for severe forms of psoriasis, but only for short periods because limited data are available. 

Long-term administration of systemic corticosteroids is associated with L.B.W., intrauterine growth restriction, gastroesophageal reflux, and even oral clefting. These drugs should be avoided in the first trimester of pregnancy, and it is recommended that at least 6 weeks lapse after delivery before their use, and to ensure 3 h between their ingestion and breastfeeding. The disease often recurs when corticosteroids are stopped, frequently in a more severe form, such as erythrodermic or pustular psoriasis [95,96,97,98,99].

Pregnant women cannot receive systemic treatment with methotrexate and acitretin because these drugs are teratogenic and contraindicated in pregnancy. Methotrexate may negatively affect embryogenesis and drive development of “fetal methotrexate syndrome” because it inhibits dihydrofolate reductase, an essential enzyme in D.N.A. synthesis. Methotrexate administration during pregnancy has been associated with large fontanelles, craniosynostosis, ocular hypertelorism, micrognathia, heart and limb reduction abnormalities, and developmental delay [100,101,102].

Fetal exposure to acitretin can cause classic retinoid syndrome, consisting of well-characterised craniofacial, cardiac, thymic, and central nervous system malformations, along with neurodevelopmental problems [101,102]. Acitretin should be discontinued at least three years before conception [103].

Apremilast, an analogue of thalidomide, is also contraindicated in pregnancy.

The treatment of psoriasis during pregnancy depends on the form of psoriasis and the severity, also on patient acceptance (Table 4). The risk/benefit ratio of each treatment will always be considered.

## 6. Biologic Treatments

Few studies have addressed biologics treatment for psoriasis in pregnancy. Information about TNF-α inhibitors arises from their use for rheumatoid arthritis and inflammatory bowel syndrome [107,108]. Currently, the following biologics can be used to treat psoriasis: adalimumab, etanercept, and infliximab. The Organisation of Teratology and Information Specialists project has found no correlation of administration of these drugs with increased risk of malformations. However, during administration, monoclonal antibody levels were similar to or even higher in umbilical cord blood than in maternal serum at term [2].

Because of these high concentrations in fetal blood, treatment with TNF-alpha inhibitors could lead to fetal and then neonatal immunosuppression. There have been several reports of fatal infections following vaccines in the first months of life (e.g., Bacillus Calmette–Guerin [108,109]. Therefore, it has been recommended to stop biologic treatments at 30 weeks of pregnancy [110,111].

Monoclonal antibodies therapy targeting cytokines specifically upregulated in psoriasis employs ustekinumab (IL-12/23 inhibitor), secukinumab (IL-17 inhibitor), and ixekizumab (IL-17 inhibitor).

Certolizumab pegol, a PEGylated, Fc-free anti-TNF agent, does not cross the placenta and should be the first-line therapy used in pregnant patients or those of child-bearing potential and psoriasis. It is the only biologic agent with clinical trial data in its label supporting potential use in pregnancy [112].

Ustekinumab is a high molecular-weight IgG1 molecule, and placental crossing is poor at the beginning of pregnancy and during the first weeks of pregnancy when organogenesis occurs [113], reducing the hypothetical teratogenic risks. Studies have demonstrated the transplacental passage of ustekinumab after 16 weeks [114]. Also, ustekinumab exhibits a long biologic half-life, slower distribution and low immune response rate. Ustekinumab is administered subcutaneously for a patient with moderate to severe psoriasis. For a weighed <100 kg one dose of 45 mg initially and four weeks later, followed by 45 mg administered subcutaneously every 12 weeks; and for a patient weighted >100 kg one dose of 90 mg initially and 4 weeks later, followed by 90 mg administered subcutaneously every 12 weeks [115].

Ustekinumab use during breastfeeding has been approved because of the very low concentration of this drug in human milk [116]. A recent report on ustekinumab use during 2012–2019 in 27 pregnancies showed that the drug was administered throughout pregnancy in four cases. Another 12 (44.44%) pregnancies were uneventful, and in one case, gestational diabetes developed. None of the patients reported birth defects or adverse events in the immediate neonatal period [117,118].

The European League Against Rheumatism task force, a multidisciplinary committee with representation from 10 European countries and the United States, has suggested that the use of ustekinumab during pregnancy, based on current evidence, is not associated with an increased rate of congenital malformations [119].

Secukinumab is a fully human monoclonal antibody that selectively targets IL-17A and shows efficacy and safety in treating moderate to severe psoriasis, including psoriasis in pregnancy and postpartum. In a report by Warren et al. on 238 mothers exposed to secukinumab during pregnancy, 65% discontinued it in the first trimester, and three continued it throughout their pregnancies. In these cases, no complications of pregnancy or fetal abnormalities attributable to the medication were reported [120].

British Association of Dermatologists guidelines indicate that certolizumab pegol should be considered the first-choice treatment in pregnant women and can be used throughout pregnancy [82]. Treatment discontinuation during pregnancy is recommended in the case of secukinumab and Ustekinumab [121].

The A.C.R. 2020 guidelines [122] include a strong recommendation for biologics treatment during lactation (treatment continuation or initiation). Table 5 summarise biological treatments in psoriasis-associated pregnancy.

## 7. Conclusions

The co-occurrence of pregnancy with different forms of psoriasis, including pustular psoriasis of pregnancy represents a high-risk situation with the potential for complications. Each case requires a tailor-made approach for the patient. Genetic and immunologic testing may be used for patients whose disease shows an unfavourable predisposition. Generalised pustular psoriasis and maternal comorbidities can add to the risk for harm. A multidisciplinary team must choose optimal treatment, taking into account fetal and maternal risks and benefits.

## Figures and Tables

**Table 1:** Psoriatic arthritis disease activity during pregnancy and postpartum.

Study	Number of Pregnancies Studied for Arthritis Activity during Pregnancy	Psoriasis Evolution during Pregnancy				Number of Pregnancies Studied for Arthritis Activity during the Postpartum Period	Psoriasis Status Postpartum		
		Improved (%)	Worsening (%)	Stable (%)	Mixed Pattern		Improved (%)	Worsening (%)	Stable (%)
Polachek et al. [17]	41	26.83 (*n* = 11)	19.51 (*n* = 8)	43.90 (*n* = 18)	9.76 (*n* = 4)	40	20 (*n* = 8)	47.50 (*n* = 19)	32.50 (*n* = 13)
Ursin et al. [37]	108	75	NS	NS		87	2	>20	NS

NS, not studied.

**Table 2:** Skin psoriatic disease activity during pregnancy and postpartum.

Study	Number of Pregnancies Studied for Skin Activity during Pregnancy	Psoriasis Evolution during Pregnancy			Number of Pregnancies Studied for Skin Activity during Postpartum Period	Psoriasis Status Postpartum		
		Improved (%)	Worsening (%)	Stable (%)		Improved (%)	Worsening (%)	Stable (%)
Polachek et al. [17]	33	32.3 (*n* = 11)	8.9 (*n* = 3)	55.9 (*n* = 19)	31	3.23 (*n* = 1)	48.39 (*n* = 15)	48.38 (*n* = 15)
Gudjonsson et al. * [11]	123	36.4	-	40		-	-	-
Murase et al. [15]	47	55.32 (*n* = 26)	23.40 (*n* = 11)	21.28 (*n* = 10)	46	8.70 (*n* = 4)	65.21 (*n* = 30)	26.08 (*n* = 12)
Mowad et al. [38]	46	35	18	46		NS	NS	NS
Park and Youn [39]	85	42 (*n* = 36)	19 (*n* = 6)	39 (*n* = 33)		NS	NS	NS
Raychudhuri [16]	91	56 (*n* = 51)	26.4 (*n* = 24)	17.6 (*n* = 16)		NS	NS	NS
Boyd et al. [19]	90	63.33 (*n* = 57)	13.33 (*n* = 12)	23.33 (*n* = 21)	90	1.11 (*n* = 1)	87.78 (*n* = 79)	11.11 (*n* = 10)
Dunna and Finlay [40]	112	41.1 (*n* = 46)	14.3 (*n* = 16)	42.9 (*n* = 48)	112 ^#^	10.70 (*n* = 12)	49.10 (*n* = 55)	36.60 (*n* = 41)

NS, not studied; * for Gudjonsson et al. we report % for patients HLA-Cw* 0602 carriers; ^#^ for Dunna and Finlay in postpartum period four patients did cannot evaluate psoriasis evolution (*n* = 4).

**Table 3:** Composite measures in PsA and clinical assessment of psoriasis outcome measures [41,42,43,44,45,46,47,48,49,50].

Composite Measures in PsA	Clinical Assessment of Psoriasis Outcome Measures
Disease Activity for Psoriatic Arthritis (D.A.P.S.A.) Psoriatic Arthritis Joint Activity Index (PsAJAI) Composite Psoriatic Disease Activity Index (C.P.D.A.I.) Bath Ankylosing Spondylitis Activity Disease Activity Index (B.A.S.D.A.I.) Ankylosing Spondylitis Disease Activity Score (A.S.D.A.S.)	Body Surface Area (B.S.A.) Psoriasis Area and Severity Index (P.A.S.I.) Physician’s Global Assessment (P.G.A.) Lattice System Physician’s Global Assessment (L.G.P.G.A.) Self-Administered P.A.S.I. (S.A.P.A.S.I.) Salford Psoriasis Index (S.P.I.)

**Table 4:** Treatment options of psoriasis during pregnancy [32,78,84,95,104,105,106].

Treatment	Administration	Observations
Anthralin	Topic	Use if clearly needed
Coal tar	Topic	Potentially mutagenic/carcinogenic (animal study data)
Calcipotriol	Topic	Use only on small surfaces when no alternatives exist
Corticosteroids	Topic	Low- to moderate-potency topical corticosteroids in short-term use is acceptable
Tacrolimus	Topic	Use only on small surfaces when no alternatives exist
Phototherapy		Can be used as adjunctive therapy in patients who show poor response to corticosteroids
Cyclosporin	Systemic	Cyclosporine is a therapeutic option for patients who are unresponsive to corticosteroids. Cyclosporine may be administered in combination or as an alternative therapy, especially first-line therapy for P.P.P.
Corticosteroids	Systemic	Use of lower dose of prednisone or prednisolone. Standard drug treatment is not considered because of the high toxicity profile and the risk of side effects in pregnant women. Corticosteroids are associated with a risk of clef palate.

P.P.P.: impetigo herpetiformis.

**Table 5:** Biological treatments in psoriasis associated pregnancy [111,112,114,120,123,124,125,126].

Biological Therapy	Observations
Certolizumab pegol	It could be the first-line therapy in pregnant patients or those of the childbearing period.
Etanercept	May cross the placenta It has generally been well-tolerated One study suggested an association between etanercept and V.A.C.T.E.R.L. syndrome.
Adalimumab	May cross the placenta No increase in the rate of miscarriage, malformations, or preterm birth.
Infliximab	No increase in adverse pregnancy outcomes for patients.
Ustekinumab	No specific risks with exposure during pregnancy or within two months prior to conception The label suggests avoidance of their use during pregnancy as a precautionary measure.
Secukinumab	No safety signals concerning spontaneous abortions or congenital malformations The label suggests avoidance of their use during pregnancy as a precautionary measure.

V.A.C.T.E.R.L. syndrome: acronym for vertebral defects, anal atresia, cardiac defects, trachea-esophageal fistula, renal anomalies, and limb anomalies.

## Data Availability

All the data are available from the corresponding author upon reasonable request.
